# Optimal use of taxanes in metastatic breast cancer

**DOI:** 10.3747/co.v16i3.377

**Published:** 2009-05

**Authors:** K.M. King, S. Lupichuk, L. Baig, M. Webster, S. Basi, D. Whyte, S. Rix

**Keywords:** Metastatic breast cancer, docetaxel, paclitaxel, nab-paclitaxel, chemotherapy

## Abstract

The role of taxanes in the treatment of breast cancer is becoming increasingly important. In clinical practice, the taxanes are now standard therapy in both early-stage and metastatic breast cancer. Since the 1990s, multiple randomized clinical trials have been evaluating the efficacy of taxanes in the treatment of metastatic breast cancer. These trials have included treatment with taxanes alone or in combination with other chemotherapeutic agents. Pre-existing published guidelines for the use of taxanes in the management of metastatic breast cancer are available. The mandate of the Alberta Cancer Board Provincial Breast Tumour Group Guideline Panel was to consider and adapt the recommendations of the existing guidelines and to develop *de novo* guidelines to account for current evidence. For this task, the panel used the adapte process, which is a systematic process of guideline adaptation developed by the adapte Collaboration.

The recommendations formulated by the panel included the identification of taxane regimens that could be offered in anthracycline-naïve patients, anthracycline-pretreated or -resistant patients, and patients overexpressing the human epidermal growth factor receptor 2. Potential toxicities and benefits in terms of time to progression, progression-free survival, overall survival, and quality of life were also considered.

## 1. INTRODUCTION

For 2007, breast cancer is projected to be the second most commonly diagnosed cancer, and the third leading cause of cancer-related mortality in Canadian women^1^. Cancer was the leading cause of potential life–years lost in Canadian adults in 2003, and breast cancer accounted for 18.3% of cancer-related premature mortality in women^1^. Approximately 6% of women with breast cancer are metastatic at diagnosis^2^, a rate that will account for just over 1300 new breast cancer diagnoses in Canadian women in 2007^1^,^2^.

Unfortunately, many women with early breast cancer will be diagnosed with a metastatic relapse in the years following treatment with locoregional and systemic adjuvant therapies. The goals of treatment in metastatic breast cancer include prolongation of survival, symptom control, and maintenance of quality of life. In Alberta, chemotherapy for breast cancer is prescribed at tertiary, associate, and community cancer clinics, all of which are affiliated with the Alberta Cancer Board.

The literature on taxanes and breast cancer has been growing exponentially since the mid-1990s. In the absence of an evidence-based provincial guideline, regional variability in taxane prescription is to be expected. The objective of creating a provincial guideline on the optimal use of taxanes in the management of metastatic breast cancer is to promote evidence-based consistency in practice and hence equitable patient access to appropriate therapies. Guidelines for taxane use in the management of meta-static breast cancer have previously been published. The guidelines published here were developed by the Alberta Cancer Board Provincial Breast Tumour Group Guideline Panel by systematically adapting the recommendations of others and by creating *de novo* recommendations to account for recent evidence.

## 2. APPROACH TO GUIDELINE DEVELOPMENT

### 2.1 Objective

The objective was to determine the optimal clinical use of taxanes in the management of metastatic breast cancer.

### 2.2 Key Clinical Questions

- Which taxane regimens can be offered to anthracycline-naïve patients with metastatic breast cancer [in which the human epidermal growth factor receptor 2 (her2) is not overexpressed]?
- Which taxane regimens can be offered to anthracycline-pretreated or -resistant patients with metastatic breast cancer (in which her2 is not overexpressed)?
- Which taxane regimens can be offered to patients with metastatic breast cancer in which her2 is overexpressed?
- What are the benefits [time to progression (ttp), progression-free survival (pfs), overall survival (os), quality of life (qol)]?
- What are the potential toxicities?

### 2.3 Target Population

The target population for the guideline is individuals with metastatic breast cancer (anthracycline-naïve or -pretreated or -resistant) who are eligible for palliative chemotherapy (hormone refractory or rapidly progressive disease, with adequate performance status and organ function).

### 2.4 Target Users

The target users for the guideline are chemotherapy-prescribing physicians, nurse practitioners, and pharmacists within the Alberta Cancer Board; the Alberta Cancer Board Pharmacy and Therapeutics Committee; and patients.

## 3. METHODS

The guideline presented here was developed by the Alberta Cancer Board Provincial Breast Tumour Group Guideline Panel using the adapte process^3^ and some aspects of the practice guidelines development cycle^4^. Panel members included 4 medical oncologists, 1 oncology pharmacist, 1 oncology nurse, and 1 methodologist. All panel members disclosed information on potential conflicts of interest before the development process started (no conflicts were reported). The Alberta Cancer Board Provincial Breast Tumour Group Guideline Panel is editorially independent of the Alberta Cancer Board.

### 3.1 Literature Search Strategy

A systematic search of medline, PubMed, cinahl, embase, CancerLit, the Cochrane Library, the Physician Data Query database, practice guideline internet sites, and conference proceedings from the American Society of Clinical Oncology (asco) and the San Antonio Breast Cancer Symposium was conducted for relevant, existing practice guidelines and other evidence. The search terms used were Taxane* Exp., Taxanes Exp., metastatic breast cancer exp., metastases, breast tumor, Women exp., adding AND/OR Anthracycline exp., Anthracyclines exp.

The search for practice guidelines was conducted for the period January 1, 2000, to August 31, 2007. Given that the most up-to-date evidence-based practice guideline selected for the adaptation process was published in 2003, the search for other evidence was conducted for the period January 1, 2003, to January 1, 2007. Other evidence cited in bibliographies and brought forward during editing and review was collected as necessary.

### 3.2 Inclusion and Exclusion Criteria

Titles and abstracts (where possible) were assessed independently by 2 reviewers for relevance. A full copy of the publication or abstract (if only a conference proceeding) was obtained if either reviewer considered the item to be relevant. These documents were assessed for inclusion and exclusion criteria as follows:

- For practice guidelines:
  1. Pertaining to individuals with metastatic (not locally advanced) breast cancer, anthracycline-naïve or pretreated
  2. Pertaining to palliative chemotherapy with reference to taxanes or taxane-containing regimens (“taxanes” are docetaxel, paclitaxel, or nab-paclitaxel)
  3. Published in the English language
  4. Clear linkage between the recommendations and the supporting literature
  5. Presentation of a comprehensive review of the relevant existing evidence with or without meta-analyses of data where appropriate
- For other evidence:
  1. Systematic review, randomized phase iii clinical trial, or randomized phase ii clinical trial reporting data on time to progression, pfs, os, or qol (with or without data on response rates)
  2. Pertaining to individuals with metastatic (not locally advanced) breast cancer, anthracycline-naïve or pretreated
  3. Pertaining to palliative chemotherapy in which a taxane or taxane-containing regimen is compared with a non-taxane chemotherapy regimen or a different taxane chemotherapy regimen (“taxanes” are docetaxel, paclitaxel, or nab-paclitaxel)
  4. Published in the English language

### 3.3 Data Synthesis

The Comprehensive Meta-analysis Package, version 2, was used for data pooling where deemed appropriate. Random effects models were used to obtain odds ratios or rate ratios.

### 3.4 External Review

A draft report was distributed for review to other members of the Alberta Cancer Board Provincial Breast Tumour Group with representation from medical oncology, nursing, and pharmacy. Reviewers were asked to read the guideline, complete a questionnaire (based on the questionnaire published in Elit *et al.*^5^), and to provide other written comments.

## 4. RESULTS

### 4.1 Literature Search

Two guidelines met the inclusion criteria and were considered relevant:

- **Cancer Care Ontario (cco)** *The Role of the Taxanes in the Management of Metastatic Breast Cancer,* Practice Guideline Report 1–3, version 2.2003 6
- **National Institute of Clinical Excellence (nice)** *Guidance on the Use of Taxanes for the Treatment of Breast Cancer,* Technology Appraisal Guidance no. 30, September 2001^7^

Four guidelines did not fully meet guideline inclusion criterion 4:

- **Scottish Intercollegiate Guidelines Network (**sign**)** *Management of Breast Cancer in Women. A National Clinical Guideline,* December 2005^8^
- **British Columbia Cancer Agency (**bcca**)** *Cancer Management Guidelines: A Guide for Women with Advanced Breast Cancer,* August 2006^9^
- **National Comprehensive Cancer Network (**nccn**)** *Invasive Breast Cancer* (“preferred chemotherapy regimens for recurrent or metastatic breast cancer”), Practice Guidelines in Oncology, v.2.2006^10^
- **Central European Cooperative Oncology Group (**cecog**)** “Second consensus on medical treatment of metastatic breast cancer,” July 2006^11^

The sign guideline was discarded because the overall recommendation that “taxanes should be considered in patients with advanced disease” was felt to be too general. The bcca, nccn, and cecog documents were not accompanied by a comprehensive literature review. Although the bcca, nccn, and cecog guidelines were not subjected to the formal adapte process, the recommendations were deliberated because the group felt that they could be influencing current local practice.

With respect to other evidence, two systematic reviews and twenty-three randomized phase ii or iii trials initially met the inclusion criteria and were considered relevant. Sixteen publications from the literature search did not meet inclusion criteria or were not considered relevant. Eight other documents (related guidelines or trials) were obtained during editing and review.

### 4.2 External Review

Five completed questionnaires were returned. Table i shows the results for the 16 closed-ended questions. Responses were favourable (“agree” or “strongly agree” on a 5-point Likert scale). Only 1 reviewer marked “neutral” to one question: “When applied, the recommendations would result in better use of resources than current usual practice.” One open-ended comment resulted in a change to the draft report. The reviewer suggested providing further explanation for the inclusion or exclusion of guidelines.

## 5. DISCUSSION

The panel considered some general principles when adapting and creating *de novo* recommendations. First, for the question of benefit, emphasis was placed on gains in os. Quality-of-life data usually did not differentiate regimens or were lacking. Options were not discarded based on toxicity data. Such data were described to allow for patient and physician preference. Second, because the optimal dose of some taxanes in some schedules has not yet been established, dose ranges reflecting those that have been studied in the various trials are presented. Deliberation of cost-effectiveness was beyond the scope of the present project.

### 5.1 Anthracycline-Naïve Patients

#### 5.1.1 Recommendation 1

If single-agent chemotherapy is preferred, an anthracycline followed sequentially by a taxane at the time of disease progression—or vice versa—is acceptable. A survival benefit has not been shown for starting with a taxane.

The following regimen is recommended:

- Docetaxel 100 mg/m^2^ every 3 weeks

Weekly taxane regimens are also a reasonable option if minimization of risk for certain toxicities associated with every-three-weeks docetaxel is desired:

- Docetaxel 35–40 mg/m^2^ weekly for 3 of every 4 weeks, or weekly for 6 of every 8 weeks
- Paclitaxel 80–90 mg/m^2^ weekly

***Recommendations from Existing Guidelines:*** The cco guideline recommends that patients who would be offered treatment with a single-agent anthracycline could also be offered single-agent docetaxel 100 mg/m^2^ every 3 weeks 6. The nice guideline does not provide guidance with respect to single-agent taxanes in anthracycline-naïve patients 7.

The bcca and cecog statements do not recommend a single-agent taxane as initial therapy in a patient who is anthracycline-naïve and who has metastatic breast cancer where her2 is not overexpressed^9^,^11^. The nccn does not differentiate anthracycline-naïve and -pretreated or -resistant patients, but suggests that a variety of single-agent taxane regimens can be considered^10^.

***Other Evidence Considered by the Panel:*** Two meta-analyses looked at the question of single-agent taxanes as compared with single-agent anthracyclines. The meta-analysis by Piccart–Gebhart *et al.* pooled individual patient data (*n* = 919) from three randomized trials^12^. The hazard ratios for the taxane as compared with the anthracycline were 1.01 [95% confidence interval (ci): 0.97 to 1.26] for survival and 1.19 (95% ci: 1.04 to 1.36) for pfs. Response rates were similar: 38% for the single-agent taxane and 33% for the single-agent anthracycline. Piccart–Gebhart *et al.*^12^ noted that there was significant heterogeneity with respect to the finding of improved pfs for the anthracycline as compared with the taxane, and that this result was largely driven by the European Organisation for the Research and Treatment of Cancer (eortc) trial^13^ that compared paclitaxel 175 mg/m^2^ every 3 weeks with doxorubicin 75 mg/m^2^ every 3 weeks. The meta-analysis by Ghersi *et al.*^13^ extracted data from published trials. The analysis by those authors of the same three trials examined by Piccart–Gebhart *et al.*^12^ found similar results. However, Ghersi *et al.*^14^ looked at ttp and did not find a difference between the taxane and anthracycline arms.

With respect to toxicities reported in the trials included in the meta-analyses, more sensory peripheral neuropathy was observed in the taxane arms, but more febrile neutropenia, mucositis, nausea and vomiting, cardiac failure, and toxic death were observed in the anthracycline arms^13^,^15^,^16^. Quality of life was analyzed in all three of the trials, and no significant differences were observed between the treatment groups with respect to physical, social, and emotional functioning, and relationship with the treating physician^13^,^15^,^16^. In one of the trials, the toxicities of doxorubicin were offset by better symptom control^13^.

In the systematic reviews, the taxanes have not been compared in subgroup analyses. In the eortc trial^13^ included in the meta-analyses, os was inferior in the paclitaxel group (15.6 months vs. 18.3 months). One phase iii trial randomized patients with anthracycline-pretreated metastatic breast cancer to receive either docetaxel 100 mg/m^2^ or paclitaxel 175 mg/m^2^ every 3 weeks^17^. Overall survival and ttp were significantly better for the docetaxel arm at the expense of greater hematologic and non-hematologic toxicities.

The panel agreed to include weekly taxane regimens as an option. The evidence and rationale are largely drawn from the anthracycline-pretreated setting and are further discussed in subsection 5.2.

***Summary Statement:*** The cco recommendation was adapted. In support of this recommendation, the panel acknowledged the results of the two meta-analyses, the individual trial data included in the meta-analyses, and one study of docetaxel compared with paclitaxel every 3 weeks (from the anthracycline-pretreated setting). Including weekly taxane regimens as an option was a *de novo* addition.

#### 5.1.2 Recommendation 2

If combination chemotherapy is preferred, non-taxane/anthracycline and taxane/anthracycline regimens are acceptable alternatives. Taxane/anthracycline combinations are superior with respect to overall response and pfs, but have not been shown to improve os. Additionally, an os benefit for using a taxane/anthracycline combination over planned sequential single-agent anthracycline followed by single-agent taxane (before disease progression or at the time of disease progression) has not been shown.

With respect to possible taxane/anthracycline regimens, doublets of docetaxel or paclitaxel plus doxorubicin or epirubicin, and the triplet docetaxel/doxorubicin/cyclophosphamide have been studied.

***Recommendations from Existing Guidelines:*** The cco guideline states that docetaxel or paclitaxel in combination with doxorubicin can be considered^6^. This recommendation contrasts with that in the nice guideline. The nice guideline evaluated data only for docetaxel/anthracycline combinations. It recommends against such combination therapy for the reason of unknown effectiveness of sequential therapy^7^.

The nccn statement lists docetaxel or paclitaxel in combination with doxorubicin as options^10^. The bcca and cecog statements do not consider taxane/anthracycline combinations as options for anthracycline-naïve patients^9^,^11^.

***Other Evidence Considered by the Panel:*** The meta-analyses by the Piccart–Gebhart and Ghersi groups also addressed the issue of taxane/anthracycline regimens as compared with non-taxane/anthracycline combination therapy^12^,^14^. Piccart–Gebhart *et al.* included individual patient data from eight randomized trials. Docetaxel was the taxane in four of those trials, and paclitaxel was the taxane in the other four. The hazard ratios for the taxane/anthracycline regimen as compared with the non-taxane/anthracycline combination were 0.95 (95% ci: 0.88 to 1.03) for os and 0.92 (95% ci: 0.85 to 0.99) for pfs.

Response rates significantly favoured the taxane/anthracycline regimens (57% vs. 46%). The authors postulated that patients with worse prognosis (visceral or estrogen receptor–negative disease) would benefit from taxane/anthracycline regimens, but this hypothesis was not supported in subgroup analyses. Ghersi *et al.*^14^ identified nine potentially eligible studies for this question, but only three studies had reported time-to-event data. Docetaxel was the taxane in one of the trials, and paclitaxel was the taxane in the other two trials. The hazard ratios for the taxane/anthracycline regimens as compared with the non-taxane combinations were 0.88 (95% ci: 0.76 to 1.02) for os and 0.81 (95% ci: 0.70 to 0.94) for ttp. Five trials reported information on response. The odds ratio for response for the taxane/anthracycline regimens as compared with the non-taxane/anthracycline combinations was 1.7 (95% ci: 1.39 to 2.08).

Four studies provided adequate data on toxicity. The taxane/anthracycline regimens were associated with significantly more leucopenia and neurotoxicity, but less nausea and vomiting. No difference in qol was observed.

Again, the systematic reviews did not compare the taxanes in subgroup analyses. One phase iii trial that compared docetaxel/doxorubicin with paclitaxel/doxorubicin has been published^18^. Outcomes were not significantly different in terms of median os (22.6 months vs. 24.1 months) and response (40% vs. 42%). More peripheral neuropathy was observed in the paclitaxel group. No difference was observed between the groups with respect to qol, although some subscores favoured the docetaxel/doxorubicin group.

The panel was also interested in the issue of taxane/anthracycline combinations as compared with sequencing of single-agent anthracycline to single-agent taxane. Two trials have examined planned sequential anthracycline followed by taxane (before progression)^19^,^20^. Alba *et al.* randomized women with metastatic breast cancer to docetaxel 75 mg/m^2^ with doxorubicin 50 mg/m^2^ every 3 weeks for 6 cycles, or doxorubicin 75 mg/m^2^ every 3 weeks for 3 cycles, followed by docetaxel 100 mg/m^2^ every 3 weeks for 3 cycles^19^. Women who were anthracycline-pretreated and randomized to the combination arm received 3 cycles of docetaxel with doxorubicin, followed by 3 cycles of docetaxel 100 mg/m^2^ every 3 weeks. No significant differences were observed for median os (21.8 months vs. 22.3 months), median ttp (9.2 months vs. 10.5 months), or overall response (51% vs. 61%). Conte *et al.* randomized participants to paclitaxel 200 mg/m^2^ with epirubicin 90 mg/m^2^ every 3 weeks for 8 cycles, or epirubicin 120 mg/m^2^ every 3 weeks for 4 cycles followed by paclitaxel 250 mg/m^2^ every 3 weeks for 4 cycles^20^. No significant differences were observed for median os (20 months vs. 26 months), median pfs (10.8 months vs. 11 months), or overall response (58.5% vs. 57.6%). Assessment of qol suggested better functioning and symptom control for the combination arm.

Sledge *et al.* were able to compare the issue of taxane/anthracycline combination with sequencing of single agents at progression^16^. The three arms in that trial were paclitaxel 150 mg/m^2^ with doxorubicin 50 mg/m^2^ every 3 weeks, paclitaxel 175 mg/m^2^ every 3 weeks followed by doxorubicin 60 mg/m^2^ every 3 weeks at progression, and doxorubicin 60 mg/m^2^ every 3 weeks followed by paclitaxel 175 mg/m^2^ every 3 weeks at progression. Just over half the participants in the single-agent arms crossed over to the alternate single agent at progression. No significant differences were found for median os between the combination arm and either sequential single-agent arm (22 months vs. 22 months vs. 18.9 months). Time to failure was significantly longer, and response rate was significantly higher for the combination arm as compared with either sequential single-agent arm.

***Summary Statement:*** The cco recommendation was adapted. In support of this recommendation, the panel acknowledged the results of two meta-analyses and one trial of docetaxel/doxorubicin compared with paclitaxel/doxorubicin. Data on taxane/anthracycline combinations compared with sequential single agents were also considered.

### 5.2 Anthracycline-Pretreated or -Resistant Patients

#### 5.2.1 Recommendation 1

If single-agent chemotherapy is preferred, a taxane regimen is recommended. Single-agent taxanes appear to improve os and response as compared with non-taxane/non-anthracycline regimens.

The following regimen is recommended:

- Docetaxel 100 mg/m^2^ every 3 weeks

Weekly taxane regimens are also a reasonable option if minimization of risk for certain toxicities associated with docetaxel every 3 weeks is desired:

- Docetaxel 35–40 mg/m^2^ weekly every 4 weeks for 3 cycles, or weekly every 8 weeks for 6 cycles
- Paclitaxel 80–90 mg/m^2^ weekly

***Recommendations from Existing Guidelines:*** The cco and nice guidelines both suggest that single-agent docetaxel and paclitaxel are options^6^,^7^. The cco guideline takes the stance that the evidence for using docetaxel every 3 weeks is more consistent and is based on larger trials than is the evidence for using paclitaxel every 3 weeks^6^. The cco suggests reserving weekly taxane regimens (options not specified) for patients who are elderly, have a low performance status, or would prefer avoiding some of the toxicities associated with taxanes every 3 weeks^6^. The nice guideline is nondirective in terms of the single-agent regimens that can be considered.

The panel noted that the recent bcca, nccn, and cecog statements also suggest that single-agent taxanes are options^9^–^11^. The bcca considers docetaxel every 3 weeks and weekly, plus paclitaxel every 3 weeks^9^. The nccn considers both docetaxel and paclitaxel every 3 weeks and weekly, plus nab-paclitaxel every 3 weeks^10^. The cecog is nondirective in terms of the single-agent regimens that can be considered^11^.

**FIGURE 1:**
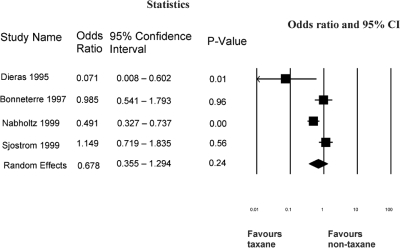
*Single-agent taxane compared with non-taxane/non-anthracycline: meta-analysis of overall survival* *21–24* *ci* *= confidence interval.*

**FIGURE 2:**
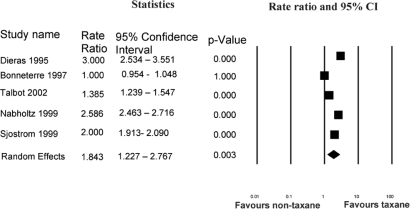
*Single-agent taxane compared with non-taxane/non-anthracycline: meta-analysis of overall response* *21–25*. *ci* *= confidence interval.*

***Other Evidence Considered by the Panel:*** Compared with non-taxane/non-anthracycline regimens, single-agent taxanes appear to improve os and response. It was possible to pool os data from four of the five studies outlined in Table ii. Figure 1 shows that, using a random-effects model, the odds ratio for survival was 0.68 (95% ci: 0.36 to 1.3) with a trend favouring the single-agent taxane. Data on overall response rate from all five studies in Table ii were pooled. Figure 2 shows that, using a random-effects model, the rate ratio for overall response was 2.23 (95% ci: 1.43 to 3.49) favouring the single-agent taxane.

Paclitaxel 175 mg/m^2^ every 3 weeks was not included as an option by the panel. First, three of the five studies in Table ii were of docetaxel compared with a non-taxane/non-anthracycline regimen. The three docetaxel studies were much larger than the two paclitaxel studies. Second, one randomized phase iii trial of docetaxel 100 mg/m^2^ compared with paclitaxel 175 mg/m^2^, both every 3 weeks, showed significantly longer median survival in the docetaxel group (15.4 months vs. 12.7 months) and significantly longer median ttp (5.7 months vs. 3.6 months)^17^. However, the docetaxel benefit came at the cost of more hematologic and non-hematologic toxicity: febrile neutropenia (14.9% vs. 1.8%), stomatitis/mucositis (10.8% vs. 0%), nausea and vomiting (8.6% vs. 2.7%), and sensory peripheral neuropathy (7.2% vs. 4.1%). Quality-of-life scores were not significantly different.

Evidence for the effectiveness—and perhaps more favourable toxicity profiles—associated with weekly taxane regimens is mounting.

***Weekly Docetaxel:*** One phase iii trial compared docetaxel 35–40 mg/m^2^ weekly every 4 weeks for 3 cycles with docetaxel 75–100 mg/m^2^ every 3 weeks^26^. No significant difference was observed with respect to median os (18.6 months vs. 18.3 months) or median pfs (5.5 months vs. 5.7 months). However, in the weekly group, overall response was lower (20.3% vs. 35.6%). Also in the weekly group, febrile neutropenia (3% vs. 10%), myalgias (3% vs. 27%), and fatigue (13.5% vs. 25%) were less frequently observed.

A randomized phase ii trial^27^ of docetaxel 40 mg/m^2^ weekly every 8 weeks for 6 cycles compared with docetaxel 100 mg/m^2^ every 3 weeks showed a longer median os for the weekly group (29.1 months vs. 20.1 months), but similar median ttp (5.7 months vs. 5.3 months) and overall response (34.1% vs. 33.3%). However, the weekly group appeared to experience less febrile neutropenia (4.9% vs. 19.5%), nausea and vomiting (12.2% vs. 14.6%), stomatitis and mucositis (7.3% vs. 17.1%), and sensory peripheral neuropathy (2.4% vs. 17.1%). In that study, slightly more asthenia and fatigue (14.6% vs. 12.2%) and more anorexia (4.9% vs. 0%) appeared to be associated with the weekly as compared with the every-three-weeks regimen.

Where reported, phase ii studies of weekly docetaxel suggest a quite variable incidence of asthenia and fatigue, ranging from 7% to 47% as summarized in the cco guideline 6.

***Weekly Paclitaxel:*** Two randomized trials have compared weekly with every-three-weeks paclitaxel. Seidman *et al.* compared paclitaxel 80 mg/m^2^ weekly with paclitaxel 175 mg/m^2^ every 3 weeks^28^. Notably, participants with her2-negative disease were also randomized to receive or not receive trastuzumab, and all participants with her2-positive disease received trastuzumab. Across the entire study population, the weekly group showed a trend to longer median os (24 months vs. 12 months), but this result was not statistically significant. Time to progression for the weekly regimen was significantly longer (9 months vs. 5 months), and overall response was significantly higher (42% vs. 29%). The incidences of febrile neutropenia (3% vs. 4%), nausea and vomiting (<5%, both arms), and stomatitis and mucositis (<5%, both arms) were similar for the treatment groups. Rates for these important toxicities are acceptably low. The incidence of grade 3 sensory peripheral neuropathy was, however, higher in the weekly group (24% vs. 12%). In cases in which her2 was not overexpressed, global qol and cancer symptom control were significantly better in the weekly group^29^.

Verrill *et al.* compared paclitaxel 90 mg/m^2^ weekly for 12 cycles with paclitaxel 175 mg/m^2^ every 3 weeks for 6 cycles^30^. No difference was detected with respect to os. The weekly group showed a trend toward longer median ttp (6 months vs. 5.5 months) and a significantly higher response rate (43% vs. 27%). Toxicity profiles of the two arms were similar.

Data from the neoadjuvant and adjuvant settings also suggest that weekly paclitaxel may be more effective than an every-three-weeks regimen. In the neoadjuvant setting, Green *et al.* examined weekly as compared with every-three-weeks paclitaxel regimens^31^. Results for the primary outcome (clinical complete response) were not statistically different. For the weekly regimen, pathologic complete response was significantly better (28.2% vs. 15.7%), as was the breast conservation rate (47% vs. 38%). Again, neurotoxicity was worse with the weekly regimen.

In women with resected high-risk node-negative or node-positive breast cancer, Eastern Cooperative Oncology Group study 1199 explored various taxane schedules (weekly vs. every-three-weeks paclitaxel and docetaxel) following a backbone of doxorubicin and cyclophosphamide^32^. The standard therapy was every-three-weeks paclitaxel. The hazard ratio for disease-free survival in patients receiving weekly paclitaxel was 1.27 (*p* = 0.006); weekly docetaxel, 1.23 (*p* = 0.02); and every-three-weeks docetaxel, 1.09 (*p* = 0.29). Weekly paclitaxel was also associated with improved survival (hazard ratio: 1.32; *p* = 0.01). In an exploratory analysis of patients with estrogen receptor–negative disease, the weekly paclitaxel and every-three-weeks docetaxel arms both proved to be superior to every-three-weeks paclitaxel in terms of disease-free survival (81.5% and 81.2% respectively vs. 76.9%). The weekly paclitaxel regimen was superior to every-three-weeks paclitaxel in terms of os (89.7% vs. 86.5%). The docetaxel groups had higher incidences of febrile neutropenia, and the weekly paclitaxel group had a significantly higher incidence of neuropathy.

***Summary Statement:*** The cco and nice recommendations for use of single-agent taxanes were adapted. More current evidence in support of recommending docetaxel over paclitaxel every 3 weeks, and further evidence in support of weekly taxane regimens, was available.

#### 5.2.2 Recommendation 2

If combination chemotherapy is preferred, taxane/non-anthracycline regimens are recommended. Compared with single-agent taxanes, taxane/non-anthracycline regimens are superior with respect to os and response. Definitive survival data for taxane/non-anthracycline combinations in comparison with sequential single-agent taxane followed by single-agent non-taxane/non-anthracycline (at progression) are not available.

The following taxane/non-anthracycline regimens should be options:

- Docetaxel 75 mg/m^2^ day 1, with capecitabine 1250 mg/m^2^ twice daily on days 1–14, every 3 weeks
- Docetaxel 75 mg/m^2^ day 1, with gemcitabine 1000 mg/m^2^ on days 1 and 8, every 3 weeks
- Paclitaxel 175 mg/m^2^ day 1, with gemcitabine 1250 mg/m^2^ days 1 and 8, every 3 weeks

***Recommendations from Existing Guidelines:*** The cco guideline includes docetaxel with capecitabine as an option in younger patients with good performance status^6^. Assessment of the other regimens (by the cco guideline examined) and of all taxane/non-anthracycline regimens (by nice) was not possible because of lack of data at the time. However, the cco has recently updated a guideline specifically on the role of gemcitabine in the management of metastatic breast cancer^33^. In that guideline, the authors conclude, based on a trial that our panel also examined, that docetaxel with gemcitabine can be considered an alternative to docetaxel with capecitabine. The cco guideline also concludes that paclitaxel with gemcitabine is an option, but that the clinical relevance questionable, given that docetaxel has been the preferred taxane for use in metastatic breast cancer in Ontario.

The panel noted that the bcca, nccn, and cecog statements also include taxane/non-anthracycline regimens as options^9^–^11^. The bcca considers docetaxel with capecitabine, or paclitaxel with gemcitabine, in the setting of an aggressive relapse in a fit patient^9^. The nccn considers the same two options^10^. The cecog states that a taxane in combination with either capecitabine or gemcitabine can be considered^11^.

***Other Evidence Considered by the Panel:*** In a large phase iii trial of docetaxel with capecitabine compared with docetaxel alone, the combination regimen was found to be superior with respect to several endpoints: median os (14.5 months vs. 11.5 months), median ttp (6.1 months vs. 4.2 months), and overall response rate (42% vs. 30%)^34^. The incidence of febrile neutropenia was similar (13% vs. 16%). As expected, the combination regimen was associated with a higher incidence of capecitabine-related toxicities. No differences between the arms were found with respect to qol.

In a large phase iii trial of paclitaxel with gemcitabine compared with paclitaxel alone, the combination regimen was found to be superior in terms of median os (18.5 months vs. 15.8 months) and median ttp (5.2 months vs. 2.9 months)^35^. With the combination regimen, the overall response rate appeared to be superior (40.8% vs. 22.1%), but this finding was not statistically significant. Clinically relevant reported toxicities appeared to be similar between the two groups: febrile neutropenia (5% vs. 1%), nausea and vomiting (2% vs. 2%), and sensory peripheral neuropathy (5% vs. 4%). Global qol was significantly better in the combination arm.

One phase iii study compared docetaxel/gemcitabine with docetaxel/capecitabine^36^. Data on os have not been presented, but no differences were observed with respect to median pfs (35 weeks vs. 35 weeks) or overall response rate (32% vs. 32%). A trend toward less febrile neutropenia was observed in the docetaxel/gemcitabine arm (8% vs. 13%). As expected, mucositis, diarrhea, and hand–foot syndrome were observed significantly less frequently in the docetaxel/gemcitabine arm.

One randomized phase ii study suggested similar outcomes for docetaxel/gemcitabine, every-three-weeks paclitaxel/gemcitabine, and weekly paclitaxel (days 1 and 8)/gemcitabine: median ttp (7.4 months vs. 7.5 months vs. 7.0 months) and overall response rate (50.3% vs. 48.6% vs. 52.3%)^37^. Data on os have not yet been reported. The incidence of febrile neutropenia appeared to be highest in the docetaxel/gemcitabine group (11.8% vs. 0% vs. 4.4%). Hence, docetaxel/gemcitabine cannot yet be recommended over docetaxel/capecitabine or paclitaxel/gemcitabine with respect to effectiveness. Docetaxel/gemcitabine could be offered in place of docetaxel/capecitabine if there is a preference to avoid capecitabine-related toxicities. Weekly paclitaxel with gemcitabine cannot yet be recommended over every-three-weeks paclitaxel with gemcitabine. Docetaxel/capecitabine have not been directly compared with paclitaxel/gemcitabine.

In the docetaxel/capecitabine^34^ versus docetaxel and the paclitaxel/gemcitabine versus paclitaxel^35^ studies, crossover from the single-agent taxane to the single-agent non-taxane/non-anthracycline at the time of progression was not planned. One randomized phase ii trial compared docetaxel 75 mg/m^2^ with capecitabine 1250 mg/m^2^ twice daily on days 1–14 every 3 weeks with docetaxel 100 mg/m^2^ every 3 weeks followed by capecitabine 1250 mg/m^2^ twice daily on days 1–14 every 3 weeks at the time of disease progression^35^. Median ttp was longer in the combination group (9.3 months vs. 7.7 months), and the overall response rate was higher (68% vs. 40%). No difference for median os was found (22 months vs. 19 months).

**FIGURE 3:**
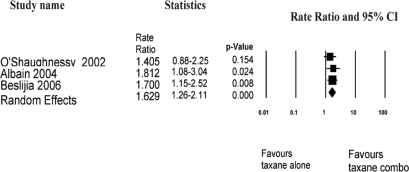
*Taxane/non-anthracycline regimen compared with single-agent taxane: meta-analysis of overall response* *34,35,38*. *ci* = *confidence interval.*

Data on overall response rate from the docetaxel/capecitabine versus docetaxel^34^, paclitaxel/gemcitabine versus paclitaxel^35^, and docetaxel/capecitabine versus sequential docetaxel to capecitabine^38^ studies, as summarized in Table iii, were pooled. Figure 3 shows that, using a random effects model, the rate ratio for overall response was 1.69 (95% ci: 1.26 to 2.11) favouring taxane/non-anthracycline combinations. Given the heterogeneous trial designs—that is, whether planned crossover occurred or did not occur in the single-agent arms—survival data were not pooled.

***Summary Statement:*** The cco recommendation for docetaxel/capecitabine was adapted. More recent evidence for docetaxel/gemcitabine and paclitaxel/gemcitabine was considered by the panel so as to create an updated recommendation on the role of taxane/non-anthracycline regimens.

### 5.3 Anthracycline-Naïve, -Pretreated, or -Resistant Patients with Corticosteroid Intolerance

#### 5.3.1 Recommendation 1

In the setting of corticosteroid intolerance, the following single-agent nanoparticle albumin-bound paclitaxel (nab-paclitaxel) regimens should be options where docetaxel or paclitaxel would otherwise be prescribed:

- Nab-paclitaxel 260–300 mg/m^2^ every 3 weeks
- Nab-paclitaxel 100–150 mg/m^2^ weekly every 4 weeks for 3 cycles

***Recommendations from Existing Guidelines:*** The cco and nice guidelines examined were unable to make recommendations regarding nab-paclitaxel because of the lack of available data at the time. However, the cco recently published a report specifically on the role of nab-paclitaxel in the treatment of metastatic breast cancer^39^. The authors concluded that women with metastatic breast cancer who are eligible for single-agent paclitaxel could be offered nab-paclitaxel as an alternative. No recommendation for specific regimens was made. The bcca and cecog did not address the role of nab-paclitaxel. The panel noted that the nccn considers nab-paclitaxel every 3 weeks to be an option^10^.

***Other Evidence Considered by the Panel:*** The panel concluded that nab-paclitaxel should be available only in the setting of corticosteroid intolerance in situations in which docetaxel or paclitaxel would otherwise be prescribed. First, without corticosteroid or antihistamine pre-medication, nab-paclitaxel is associated with hypersensitivity reactions (incidence: <1%)^40^. Second, despite the low incidence of hypersensitivity reactions without pre-medication, the safety of nab-paclitaxel after a demonstrated hypersensitivity reaction to docetaxel or paclitaxel has not been proved, and hence, nab-paclitaxel cannot currently be recommended in that setting. Finally, in the absence of definitive data showing improved os or significantly reduced toxicities for nab-paclitaxel as compared with any of the solvent-based taxane regimens, the panel felt that nab-paclitaxel should not be routinely offered as a single-agent option.

One randomized phase ii study compared three nab-paclitaxel arms (300 mg/m^2^ every 3 weeks, 100 mg/m^2^ weekly for 3 of 4 weeks for 3 cycles, and 150 mg/m^2^ weekly for 3 of 4 weeks for 3 cycles) and one docetaxel arm (100 mg/m^2^ every 3 weeks)^41^. All three nab-paclitaxel arms showed reduced hazard for disease progression as compared with the every-three-weeks docetaxel arm. The pfs curve for nab-paclitaxel 150 mg/m^2^ weekly appeared to be the most favourable. In the weekly study arms, response was significantly higher than it was in either the every-three-weeks nab-paclitaxel arm or the docetaxel arm. The incidence of febrile neutropenia was 1% in all three nab-paclitaxel arms, and 7% in the docetaxel arm. The incidence of sensory peripheral neuropathy was lowest in the nab-paclitaxel 100 mg/m^2^ weekly arm and the docetaxel arm (7% and 5% respectively). In the nab-paclitaxel 150 mg/m^2^ weekly arm and the every-three-weeks nab-paclitaxel arm, the incidences of sensory peripheral neuropathy were 12% and 14%.

In a phase iii trial of nab-paclitaxel 260 mg/m^2^ compared with paclitaxel 175 mg/m^2^, the nab-paclitaxel group showed a significantly longer median ttp (23 months vs. 16.9 months) and a trend toward longer median os (65 weeks vs. 55.7 weeks)^40^. The incidence of sensory peripheral neuropathy in the nab-paclitaxel group was 10%, and in the paclitaxel group, it was 2%.

***Summary Statement:*** The panel largely considered the relevant trials of nab-paclitaxel compared with the other taxanes to create a *de novo* recommendation.

### 5.4 Anthracycline-Naïve, -Pretreated, or -Resistant Patients in Whom HER2 Is Overexpressed

#### 5.4.1 Recommendation 1

Up front, a taxane/trastuzumab combination is recommended. The addition of trastuzumab to a taxane has been shown to improve os and response. Although the addition of trastuzumab to anthracycline regimens has also been shown to improve os and response, the incidence of cardiac failure is unacceptable. The addition of carboplatin to taxane/trastuzumab combinations has not yet been shown to improve os or to consistently increase response.

The strongest evidence is for the following single-agent taxane regimens, plus trastuzumab:

- Docetaxel 100 mg/m^2^ every 3 weeks
- Paclitaxel 175 mg/m^2^ every 3 weeks

***Recommendations from Existing Guidelines:*** The cco and nice guidelines examined did not make specific recommendations for patients with metastatic breast cancer in which her2 is overexpressed.

The cco published a separate guideline on the role of trastuzumab in the treatment of women with her2-overexpressing metastatic breast cancer^42^. Either docetaxel or paclitaxel every 3 weeks, plus trastuzumab, is recommended as first-line therapy.

The panel noted that, in this setting, the bcca and nccn statements recommended the addition of trastuzumab to either single-agent docetaxel or paclitaxel, or to paclitaxel/carboplatin^9^,^10^ The cecog statement recommended the addition of trastuzumab to a non-anthracycline regimen, which could include a taxane^11^.

***Other Evidence Considered by the Panel:*** A randomized phase ii study compared docetaxel alone with docetaxel 100 mg/m^2^ every 3 weeks plus weekly trastuzumab^43^. More than half the participants had been exposed to an anthracycline in the adjuvant setting. The addition of trastuzumab improved median ttp (11.7 months vs. 6.1 months), median os (31.2 months vs. 22.7 months), and overall response rate (61% vs. 34%).

A large phase iii study compared paclitaxel alone with paclitaxel 175 mg/m^2^ every 3 weeks plus weekly trastuzumab in the anthracycline pre-treated setting, and doxorubicin/cyclophosphamide alone with doxorubicin/cyclophosphamide every 3 weeks plus weekly trastuzumab in the anthracycline-naïve setting^44^. The addition of trastuzumab to paclitaxel improved median ttp (6.9 months vs. 3.0 months), median os (22.1 months vs. 18.4 months), and overall response rate (49% vs. 17%). The addition of trastuzumab to doxorubicin/cyclophosphamide also improved median os (26.8 months vs. 21.4 months), but the cardiac event rate was unacceptably high (28% vs. 9.6%).

Finally, a randomized phase ii study examined weekly paclitaxel 80 mg/m^2^ plus or minus weekly trastuzumab^45^. Women with advanced breast cancer and 2+ or 3+ overexpression of her2 by immunohistochemistry (ihc) were included. For the subgroup with ihc 3+ (84 of 124), median ttp was significantly longer (369 days vs. 272 days), and response was significantly higher (75% vs. 56.9%) for those who received paclitaxel plus trastuzumab.

The addition of carboplatin to taxane/trastuzumab doublets has not yet been shown to improve os or to consistently improve overall response rates. One phase iii trial compared docetaxel/trastuzumab/carboplatin with docetaxel/trastuzumab^46^. Median ttp was similar in the two arms (10.4 months vs. 11.1 months), as was overall response rate (73% vs. 73%). Median os was 41.7 months for the triplet arm, but had not yet been reached in the doublet arm.

One phase iii trial compared paclitaxel/trastuzumab/carboplatin with paclitaxel/trastuzumab^47^. Median pfs was longer in the triplet arm (10.7 months vs. 7.1 months), and the overall response rate was higher (52% vs. 36%). No difference was found for median os (35.7 months vs. 32.2 months). Both regimens were well tolerated, although a higher rate of grade 4 febrile neutropenia was observed in the carboplatin/paclitaxel/trastuzumab arm.

A protocol for a Cochrane Collaboration systematic review examining the efficacy of trastuzumab plus other drug therapy, or trastuzumab alone in her2-positive metastatic breast cancer is still pending.

***Summary Statement:*** The panel largely considered the relevant trials of taxane regimens plus or minus trastuzumab in metastatic breast cancer in which her2 is overexpressed to create a *de novo* recommendation.

## 5. CONCLUSIONS

The benefits of taxane chemotherapy in metastatic breast cancer have been well established. The goal of the present initiative was to provide evidence-based guidelines for the use of taxanes in patients with metastatic breast cancer. These guidelines have been developed through systematic adaptation of existing guidelines and the creation of *de novo* guidelines. The 6 recommendations that were formulated will be updated annually as new evidence becomes available. The panel acknowledges that many patients will have received taxanes in the adjuvant setting, and the decision regarding whether the patient’s disease is refractory to taxane treatment is left to the discretion of the physician providing treatment.

### 5.1 Limitations

The major limitation encountered in using the adapte process was that the two evidence-based guidelines selected, from nice^7^ and cco^6^, were released in 2001 and 2003 respectively. A significant amount of evidence that was more current was reviewed to ensure up-to-date recommendations. In some instances, *de novo* recommendation development was required.

### 5.2 Implications for the Alberta Cancer Board

Most regimens recommended or listed as options in the present guideline were available through the Alberta Cancer Board Outpatient Drug Benefit Program (odbp) as of July 2007 when the draft report was being sent for external review. Docetaxel/gemcitabine would require a formulary addition request to the provincial Pharmacy and Therapeutics Committee (which takes into account cost implications). Nab-paclitaxel has been added to the odbp. In contrast to the present guideline, odbp criteria for use of nab-paclitaxel includes “patients who have had severe acute infusion reactions with paclitaxel or docetaxel considered by the treating physician to be due to the vehicle of the taxanes” or “patients who have experienced severe toxicity from previous administration of other taxanes.”

### 5.3 Implementation

The final draft of the present guideline has been posted on the Alberta Cancer Board Web site and distributed to all target users.

### 5.4 Scheduled Review and Update

The Alberta Cancer Board Provincial Breast Tumour Group Guideline Panel will meet annually to review new guidelines and other pertinent evidence. The present guideline will be updated accordingly, and target users will be notified.

## Figures and Tables

**TABLE I:** External review questionnaire

*Question*	*Response frequency*		
	*Neutral*	*Agree*	*Strongly agree*
The guideline panel is credible.		1	4
The guideline is unlikely to be influenced by vested interests.		2	3
Rationale for developing the guideline is clear.		2	3
There is a need for a provincial guideline on this topic.		2	3
The literature search is relevant and complete.		1	4
I agree with the methodology used for summarizing the evidence.		1	4
The results are interpreted according to my understanding of data.		2	3
The draft recommendations are clear.		1	4
The draft recommendations are reasonable.		2	3
When applied, the recommendations will produce more benefit than harm for patients.		1	4
The recommendations are suitable for the intended patients.		2	3
The draft report presents options that will be acceptable to patients.		1	4
When applied, the recommendations would result in better use of resources than current usual practice.	1	2	2
Following the recommendations would not require reorganization of services in my practice setting.		2	3
The draft recommendations are likely to be supported by most of my colleagues.		3	2
The draft report should be approved as practice guideline.		3	2

**TABLE II:** Single-agent taxane compared with non-taxane/non-anthracycline regimens

*Reference*	*Phase*	*Treatment arms*	*Pts (n)*	*Medianpfs(months)*	*Medianttp(months)*	*Medianos(months)*	*orr(%)*
Bonneterre *et al.,* 1997^21^	iii	Docetaxel	86	nr	6.5	16	43
		Vinorelbine/5-fu	90	nr	5.1	15	38.8
Nabholtz *et al.,* 1999^22^	iii	Docetaxel	203	16^a^	19^a^	11.4^a^	30^a^
		Mitomycin/vinblastine	189	10	11	8.7	11.6
Sjöström *et al.,* 1999^23^	iii	Docetaxel	143	nr	6.3^a^	10.4	42^a^
		Methotrexate/5-fu	139	nr	3.0	11	21
Dieras *et al.,* 1995^24^	ii	Paclitaxel	36	9.1	3.5^b^	12.7	15
		Mitomycin	36	6.7	1.6	8.4	5
Talbot *et al.,* 2002^25^	ii	Paclitaxel	19	nr	3.1	9.4	36
		Capecitabine	22	nr	3.0	7.6	26

^a^ *p* < 0.01.

^b^ *p* < 0.05.

Pts = patients; pfs = progression-free survival; ttp = time to progression; os = overall survival; orr = overall response rate; nr = (not reported); 5-fu = 5-fluorouracil.

**TABLE III:** Taxane/non-anthracycline regimen compared with single-agent taxane

*Reference*	*Phase*	*Treatment arms*	*Pts (n)*	*Median pfs(months)*	*Medianttp(months)*	*Medianos(months)*	*orr(%)*
O’Shaughnessy *et al.,* 2002^34^	iii	Docetaxel/capecitabine	255	nr	6.1^a^	14.5^b^	42^a^
		Docetaxel	256	nr	4.2	11.5	30
Albain *et al.,* 2004^35^ (abstract)	iii	Paclitaxel/gemcitabine	267	nr	5.2^b^	18.5^b^	40.8
		Paclitaxel	262	nr	2.9	15.8	22.1
Beslija *et al.,* 2006^38^ (abstract)	ii	Docetaxel/capecitabine	50	nr	9.3^b^	22	68^a^
		Docetaxel to capecitabine at progression	50	nr	7.7	19	40

^a^ *p* < 0.01.

^b^ *p* < 0.05.

Pts = patients; pfs = progression-free survival; ttp = time to progression; os = overall survival; orr = overall response rate; nr = not reported; 5-fu = 5-fluorouracil

## References

[1] Canadian Cancer Society and the National Cancer Institute of Canada (2007). Canadian Cancer Statistics 2007.

[2] Ries LAG, Melbert D, Krapcho M (2007). seer Cancer Statistics Review 1975–2004 [Web resource]. seer.cancer.gov/csr/1975_2004/.

[3] The adapte Collaboration Manual for Guideline Adaptation.

[4] Browman GP, Levine MN, Mohide EA (1995). The practice guidelines development cycle: a conceptual tool for practice guidelines development and implementation. J Clin Oncol.

[5] Elit L, Zitzelsberger L, Fung-Kee-Fung M (2007). Use of systemic therapy in women with recurrent ovarian cancer—development of a national clinical practice guideline. Gynecol Oncol.

[6] Verma S, Trudeau M, Pritchard K, Oliver T, and the members of the Breast Cancer Disease Site Group (2003). The Role of the Taxanes in the Management of Metastatic Breast Cancer.

[7] United Kingdom, National Institute for Health and Clinical Excellence (nice) (2001). Guidance on the Use of Taxanes for the Treatment of Breast Cancer. Technology appraisal guidance no. 30.

[8] Scottish Intercollegiate Guidelines Network (sign) (2005). Management of Breast Cancer in Women A National Clinical Guideline.

[9] BC Cancer Agency (2007). Cancer Management Guidelines: A Guide for Women with Advanced Breast Cancer.

[10] National Comprehensive Cancer Network (nccn) (2007). Invasive Breast Cancer.

[11] Beslija S, Bonneterre J, Burstein H (2007). Second consensus on medical treatment of metastatic breast cancer. Ann Oncol.

[12] Piccart–Gebhart MJ, Burzykowski T, Buyse M (2008). Taxanes alone or in combination with anthracyclines as first-line therapy of patients with metastatic breast cancer. J Clin Oncol.

[13] Paridaens R, Biganzoli L, Bruning P (2000). Paclitaxel versus doxorubicin as first-line single-agent chemotherapy for meta-static breast cancer: a European Organization for Research and Treatment of Cancer Randomized Study with cross-over. J Clin Oncol.

[14] Ghersi D, Wilcken N, Simes J, Donoghue E (2005). Taxane containing regimens for metastatic breast cancer. Cochrane Database Syst Rev.

[15] Chan S, Friedrichs K, Noel D (1999). Prospective randomized trial of docetaxel versus doxorubicin in patients with metastatic breast cancer. J Clin Oncol.

[16] Sledge GW, Neuberg D, Bernardo P (2003). Phase iii trial of doxorubicin, paclitaxel, and the combination of doxorubicin and paclitaxel as front-line chemotherapy for metastatic breast cancer: an Intergroup trial (E1193). J Clin Oncol.

[17] Jones SE, Erban J, Overmoyer B (2005). Randomized phase iii study of docetaxel compared with paclitaxel in metastatic breast cancer. J Clin Oncol.

[18] Cassier PA, Chabaud S, Trillet–Lenoir V (2008). A phase-iii trial of doxorubicin and docetaxel versus doxorubicin and paclitaxel in metastatic breast cancer: results of the erasme 3 study. Breast Cancer Res Treat.

[19] Alba E, Martín M, Ramos M (2004). Multicenter randomized trial comparing sequential with concomitant administration of doxorubicin and docetaxel as first-line treatment of metastatic breast cancer: a Spanish Breast Cancer Research Group (geicam-9903) phase iii study. J Clin Oncol.

[20] Conte PF, Guarneri V, Bruzzi P (2004). Concomitant versus sequential administration of epirubicin and paclitaxel as first-line therapy in metastatic breast carcinoma: results for the Gruppo Oncologico Nord Ovest randomized trial. Cancer.

[21] Bonneterre J, Roche H, Monnier A (1997). Taxotere (txt) versus 5-fluorouracil + navelbine (fun) as second-line chemotherapy (ct) in patients (pts) with metastatic breast cancer (mbc) (preliminary results) [abstract 564]. Proc Am Soc Clin Oncol.

[22] Nabholtz JM, Senn HJ, Bezwoda WR (1999). Prospective randomized trial of docetaxel versus mitomycin plus vinblastine in patients with metastatic breast cancer progressing despite previous anthracycline-containing chemotherapy. 304 Study Group. J Clin Oncol.

[23] Sjöström J, Blomqvist C, Mouridsen H (1999). Docetaxel compared with sequential methotrexate and 5-fluorouracil in patients with advanced breast cancer after anthracycline failure: a randomised phase iii study with crossover on progression by the Scandinavian Breast Group. Eur J Cancer.

[24] Dieras V, Marty M, Tubiana N (1995). Phase ii randomized study of paclitaxel versus mitomycin in advanced breast cancer. Semin Oncol.

[25] Talbot DC, Moiseyenko V, Van Belle S (2002). Randomised, phase ii trial comparing oral capecitabine (Xeloda) with paclitaxel in patients with metastatic/advanced breast cancer pre-treated with anthracyclines. Br J Cancer.

[26] Rivera E, Mejia JA, Arun B (2006). Phase iii study of docetaxel weekly (dw) versus every 3 weeks (d) in patients with metastatic breast cancer: final results [abstract 574]. Proc Am Soc Clin Oncol.

[27] Tabernero J, Climent MA, Lluch A (2004). A multicentre, randomised phase ii study of weekly or 3-weekly docetaxel in patients with metastatic breast cancer. Ann Oncol.

[28] Seidman AD, Berry D, Cirrincione C (2008). Randomized phase iii trial of weekly compared with every-three-weeks paclitaxel for metastatic breast cancer, with trastuzumab for all her-2 overexpressors and random assignment to trastuzumab or not in her-2 nonoverexpressors: final results of Cancer and Leukemia Group B protocol 9840. J Clin Oncol.

[29] Naughton MJ, Gu L, Wang XF, Seidman AD, Winer E, Kornblith AB (2006). Quality of life (qol) companion to calgb 9840: a phase iii study of paclitaxel (*p*) via weekly 1 hour (hr) versus standard 3 hour infusion every 3 weeks with trastuzumab in the treatment of patients with/without her-2/*neu*-overexpressing metastatic breast cancer [abstract 674]. Proc Am Soc Clin Oncol.

[30] Verrill MW, Lee J, Cameron DA (2007). Anglo–Celtic iv: first results of a UK National Cancer Research Network randomised phase 3 pharmacogenetic trial of weekly versus 3 weekly paclitaxel in patients with locally advanced or metastatic breast cancer (abc) [abstract LBA1005]. Proc Am Soc Clin Oncol.

[31] Green MC, Buzdar AU, Smith T (2005). Weekly paclitaxel improves pathologic complete remission in operable breast cancer when compared with paclitaxel once every 3 weeks. J Clin Oncol.

[32] Sparano JA, Wang M, Martino S (2008). Weekly paclitaxel in the adjuvant treatment of breast cancer. N Engl J Med.

[33] Dent S, Messersmith H, Trudeau M, the Breast Cancer Disease Site Group (2007). The Role of Gemcitabine in the Management of Metastatic Breast Cancer: A Clinical Practice Guideline. Evidence-based series 1–12.

[34] O’Shaughnessy J, Miles D, Vukelja S (2002). Superior survival with capecitabine plus docetaxel combination therapy in anthracycline-pretreated patients with advanced breast cancer: phase iii trial results. J Clin Oncol.

[35] Albain KS, Nag S, Calderillo–Ruiz G (2004). Global phase iii study of gemcitabine plus paclitaxel (gt) vs. paclitaxel (t) as frontline therapy for metastatic breast cancer (mbc): first report of os [abstract 510]. Proc Am Soc Clin Oncol.

[36] Chan S, Romieu G, Huober J (2009). Phase iii study of gemcitabine plus docetaxel compared with capecitabine plus docetaxel for anthracycline-pretreated patients with metastatic breast cancer. J Clin Oncol.

[37] Khoo KS, Manzoor Zaidi SH, Srimuninnimit V (2006). Gemcitabine and split-dose paclitaxel or docetaxel in metastatic breast cancer: a randomised phase ii study. Eur J Cancer.

[38] Beslija S, Obralic N, Basic H (2006). Randomized trial of sequence vs. combination of capecitabine (x) and docetaxel (t): xt vs. t followed by x after progression as first-line therapy for patients (pts) with metastatic breast cancer (mbc) [abstract 571]. Proc Am Soc Clin Oncol.

[39] Hamm C, Walter–Dilks C (2007). The Role of Albumin-Bound Paclitaxel (Abraxane) in the Treatment of Metastatic Breast Cancer. www.cancercare.on.ca/pdf/pebcced6s.pdf.

[40] Gradishar WJ, Tjulandin S, Davidson N (2005). Phase iii trial of nanoparticle albumin-bound paclitaxel compared with poly-ethylated castor oil-based paclitaxel in women with breast cancer. J Clin Oncol.

[41] Gradishar W, Krasnojon D, Cheporov S (2007). Randomized comparison of weekly or every-3-week (q3w) nab-paclitaxel compared to q3w docetaxel as first-line therapy in patients (pts) with metastatic breast cancer (mbc) [abstract 1032]. Proc Am Soc Clin Oncol.

[42] Crump M, Trudeau M, Sinclair S, O’Mally F, and members of the Breast Cancer Disease Site Group (2005). *The Role of Trastuzumab (Herceptin) in the Treatment of Women with her2/*neu-*Overexpressing Metastatic Breast Cancer*.

[43] Marty M, Cognetti F, Maraninchi D (2005). Randomized phase ii trial of the efficacy and safety of trastuzumab combined with docetaxel in patients with human epidermal growth factor receptor 2–positive metastatic breast cancer administered as first-line treatment: the M77001 study group. J Clin Oncol.

[44] Slamon DJ, Leyland–Jones B, Shak S (2001). Use of chemotherapy plus a monoclonal antibody against her2 for metastatic breast cancer that overexpresses her2. N Engl J Med.

[45] Gasparini G, Gion M, Mariani L (2007). Randomized phase ii trial of weekly paclitaxel alone versus trastuzumab plus weekly paclitaxel as first-line therapy of patients with her-2 positive advanced breast cancer. Breast Cancer Res Treat.

[46] Forbes JF, Kennedy J, Pienkowski T (2006). bcirg 007: randomized phase iii trial of trastuzumab plus docetaxel with or without carboplatin first line in her2 positive metastatic breast cancer (mbc) [abstract LBA516]. Proc Am Soc Clin Oncol.

[47] Robert N, Leyland–Jones B, Asmar L (2006). Randomized phase iii study of trastuzumab, paclitaxel, and carboplatin compared with trastuzumab and paclitaxel in women with her-2-overexpressing metastatic breast cancer. J Clin Oncol.

